# Disturbance rejecting PID-FF controller design of a non-ideal buck converter using an innovative snake optimizer with pattern search algorithm

**DOI:** 10.1016/j.heliyon.2024.e34448

**Published:** 2024-07-14

**Authors:** Cihan Ersali, Baran Hekimoglu, Musa Yilmaz, Alfredo A. Martinez-Morales, Tahir Cetin Akinci

**Affiliations:** a Department of Electrical and Electronics Engineering, Batman University, Batman, 72100, Turkey; b Bourns College of Engineering, Center for Environmental Research and Technology, University of California at Riverside, Riverside, CA, 92521, USA; c Winston Chung Global Energy Center, University of California at Riverside, Riverside, CA, 92521, USA; d Electrical Engineering Department, Istanbul Technical University, 34469, Istanbul, Turkey; e Electrical and Computer Engineering Department, University of California at Riverside, Riverside, CA, 92521, USA

**Keywords:** Disturbance rejection, Non-ideal buck converter, Pattern search, PID-FF controller, Pole placement, Opposition-based learning, Snake optimizer algorithm

## Abstract

The optimal design of a proportional-integral-derivative controller with two cascaded first-order low-pass filters (PID-FF) for non-ideal buck converters faces significant challenges, including effective disturbance rejection, robustness to parameter variations, and the mitigation of high-frequency signal noise, with existing approaches often struggling and leading to suboptimal performance in practical applications. This study addresses these challenges by introducing a constraint on the open-loop crossover frequency to mitigate high-frequency noise and ensuring the controller prioritizes maintaining constant output voltage and robust responsiveness to input voltage and load current variations. This study also introduces an innovative metaheuristic algorithm, the opposition-based snake optimizer with pattern search (OSOPS), designed to address these limitations. OSOPS enhances the Snake Optimizer (SO) by integrating opposition-based learning (OBL) and Pattern Search (PS), thereby improving its exploration and exploitation capabilities. The proposed algorithm design includes a crossover frequency constraint aimed at counteracting high-frequency noise and ensuring robust performance under diverse disturbances. The efficacy of the OSOPS algorithm is demonstrated through rigorous statistical box plot analysis and convergence response comparisons with the original SO algorithm. Additionally, we systematically compare the performance of the OSOPS-based PID–FF–controlled non-ideal buck converter system against systems utilizing the original SO algorithm and the classical pole placement (PP) method. This evaluation encompasses transient and frequency responses, disturbance rejection, and robustness analysis. The results reveal that the OSOPS-based system outperforms the SO- and PP-based systems with 14.21 % and 32.10 % faster rise times, along with 15.38 % and 84.95 % faster settling times, respectively. The OSOPS and SO systems also exhibit higher bandwidths, exceeding the PP-based system by 18.74 % and 17.03 %, respectively. By addressing the key challenges in PID-FF controller design for non-ideal buck converters, this study provides a substantial advancement in control strategy, promising enhanced performance in practical applications.

## 1 Introduction

The enhancement of energy transfer efficiency, performance, and reliability in power electronics devices, ranging from power delivery modules in mainboards for smartwatches to high-performance desktop computers, heavily relies on the DC-DC power conversion [[1], [2], [3]]. Critical aspects of these devices include power conversion efficiency and the capacity to maintain stable voltage regulation [4,5]. Additionally, regulated DC power delivery is essential for the optimal operation of battery-powered vehicles and everyday portable electronic devices. The DC-DC buck converter, embedded in the circuitry responsible for delivering power to these devices, becomes crucial. However, a practical approach requires considering non-idealities, incorporating elements like internal resistances of capacitors and inductors inherent in real-world circuits [[6], [7], [8]]. Incorporating a controller capable of handling these complexities is imperative for achieving a robust, efficient buck converter with favorable steady-state, dynamic behavior, and fast transient response.

The intrinsic nature of a switching converter and its nonlinear structure pose a considerable challenge in designing an effective controller for a buck converter [9,10]. Consequently, numerous studies have explored different controller types to devise control strategies for DC converters. Some widely employed and extensively studied controllers in the literature include sliding-mode control [11], Proportional Integral Derivative (PID) control [12], adaptive control [13], fractional order control [14], fuzzy logic control [15], model predictive control [16], and neural network-based control [17], etc. PID control stands out as one of the most popular options among these options. It finds widespread use in controlling power electronic converters and industrial applications due to its relatively straightforward parameter tuning and simple implementation in large systems [18].

While PID controllers offer desirable advantages and excel in systems resistant to measurement noise, they prove insufficient in applications demanding higher precision across a broader frequency range [19,20]. Additionally, PID controllers struggle to handle unexpected conditions effectively, such as rapid load changes, input voltage changes, and parametric fluctuations. In contrast, advanced versions of PID controllers have the potential to deliver superior performance [21,22]. A PID-FF, also known as a type 3 controller, emerges as a promising solution to address the shortcomings of conventional PIDs. It features two additional low-pass filters, effectively reducing sensitivity to measurement noise induced by the derivative part of PID and increasing phase and gain margins [23,24]. The additional parameters can be optimized alongside other PID parameters or selected as predetermined values aligned with the designer's desired frequency response as in the PP design method. These advantages are achieved without compromising stability margins and the performance of the low-frequency band [25,26].

A crucial aspect of controller design is the parameter tuning process, as the attainment of design requirements such as overshoot, settling time, phase, gain margins, and robustness to parameter variations heavily depend on this process. Numerous studies in the literature have explored various parameter-tuning methods for PID controllers [[27], [28], [29], [30]]. While these methods can yield sufficiently good parameters for controllers with acceptable performance, it has been demonstrated that their ability to identify the optimal parameters is inadequate compared to the capabilities of more advanced metaheuristic algorithms [[31], [32], [33], [34], [35]].

Extensive investigations have been carried out in the realm of power electronics and power conversion systems to optimize controller parameters. Diverse metaheuristic algorithms, such as atom search optimization (ASO) [36], weIghted meaN oF vectOrs optimizer (INFO) [37], hunger games search (HGS) optimizer [38], Aquila optimizer (AO) [39], particle swarm optimization [40], manta-ray foraging optimizer (MRFO) [41], chimp optimization algorithm (ChOA) [[42], [43], [44]], marine predators algorithm (MPA) [45], fuzzy whale optimization algorithm (FWOA) [46], grey wolf optimizer (GWO) [47], snow ablation optimizer (SAO) [48], and gorilla troops optimizer (GTO) [49] have been employed for this purpose. Snake optimizer (SO) [50] is a relatively new algorithm with limited utilization in power electronic converters and control in the literature at the time of writing this article. In Ref. [51], an improved version of the snake optimizer is proposed to optimize the capacity of a hybrid energy storage system in a wind turbine application.

The studies highlighted above clearly indicate that the application of metaheuristic algorithms to controller parameter tuning yields promising results, contributing to the development of highly effective systems. Nevertheless, like many other metaheuristic algorithms discussed, they encounter certain disadvantages that hinder them from realizing their full potential. These drawbacks can be mitigated or eliminated by incorporating supplementary algorithms. Consequently, this study introduces a new metaheuristic algorithm, Opposition-Based Snake Optimizer, with pattern search (OSOPS). Representing an enhanced snake optimizer (SO), OSOPS integrates opposition-based learning (OBL) and pattern search (PS) to enhance the SO's exploration and exploitation capabilities. Applying this methodology to the parameter tuning of the PID-FF controller for a non-ideal buck converter system provides optimal parameters, facilitating the development of a more efficient, responsive, robust, and stable system.

When implementing the OSOPS algorithm for the non-ideal buck converter system, addressing high-frequency noise in the output voltage is crucial. To mitigate this noise, a constraint on the open-loop crossover frequency (6–12 kHz) is introduced in the optimization problem. Although parameters outside this range might offer better performance, they are disregarded to ensure practical and effective designs, particularly for disturbance rejection and signal attenuation. The chosen frequency range allows fair comparison with systems using classical PP methods and can be extended to 20 kHz. The controller, designed as a voltage regulator, focuses on maintaining a stable output voltage and responding robustly to input voltage and load current changes, improving disturbance rejection and system responsiveness.

Consequently, this study introduces a novel metaheuristic algorithm called OSOPS, an enhanced version of the SO. It significantly improves exploration and exploitation capabilities by incorporating OBL and PS. OBL diversifies the search process by considering opposite solutions and encouraging exploration in different regions of the search space, while PS refines candidate solutions locally, ensuring precise optimization. This combined approach enhances the algorithm's performance, as demonstrated through superior convergence response and statistical box plot analyses. OSOPS effectively tunes PID-FF controller parameters for a non-ideal buck converter system, optimizing for efficient load current disturbance rejection and making it suitable for real-time applications. To address potential high-frequency noise in the output voltage due to the switching nature of power electronics circuits, the aforementioned constraint on the open-loop crossover frequency is introduced, ensuring practical design and superior performance within the specified frequency range.

### 1.1 Contributions

Following is the contribution summary of this study.

1. A novel metaheuristic algorithm, OSOPS, is introduced, and an enhanced SO is achieved by incorporating OBL and PS. This modification improves the original algorithm's exploration and exploitation capabilities.
2. Demonstrating the superiority of the OSOPS algorithm over the original SO algorithm through convergence response and statistical box plot analyses.
3. Pioneering the application of both the SO and the proposed OSOPS algorithms to tune the parameters of the PID-FF controller for a non-ideal buck converter system.
4. The algorithm is designed to optimize the PID-FF controller parameters to efficiently reject load current disturbances within the system, which is more suitable for real-time applications.
5. Conducting comprehensive testing on the OSOPS-based PID–FF–controlled non-ideal buck converter system, including disturbance rejection evaluations for input voltage and load current changes, transient response, and frequency response analyses. Additionally, robustness analysis was performed, considering component degradation of both main and parasitic elements, and compared with results obtained using the PP method and SO algorithm.

## 2 Materials and methods

### 2.1 Snake optimizer

Snake optimizer (SO) is a metaheuristic algorithm inspired by the mating behavior of snakes. In the natural context, snakes engage in mating activities when the temperature drops and food is abundant; otherwise, they focus on foraging or consuming available food. Consequently, the algorithmic process comprises two distinct stages during the search process: exploration and exploitation. Exploration occurs in environments lacking food and characterized by cold temperatures, prompting snakes to search for sustenance. Transitioning into the exploitation phase, snakes undergo various stages to enhance efficiency. When food is available, and the temperature is high, snakes prioritize feeding. In contrast, if the temperature is cold and food is present, snakes initiate the mating process. Mating involves two modes: fight mode, where males compete for females and females choose their mates, and mating mode, where pairs mate based on food availability. In suitable environments, mating may lead to the laying of eggs by the female, eventually hatching into new snakes [50].

#### 2.1.1 Mathematical model

##### 2.1.1.1 Initialization

Like other metaheuristic algorithms, the SO algorithm initiates the optimization process by creating a random population that is uniformly distributed. The initial population can be acquired using Eq. (1).

(1) $$X_{i}=X_{\min}+rand\left(X_{\max}-X_{\min}\right)$$

here, $X_{i}$ denotes the position of the $i^{th}$ individual, *rand* is a randomly generated number within the range of 0–1, and $X_{\min}$ and $X_{\max}$ are the lower and upper bounds of the problem, respectively.

Assuming an equal distribution between males and females, each group constitutes 50 % of the overall population. Subsequently, the population is divided into two categories: males and females. This division is accomplished through Eqs. (2), (3), where *N*, $N_{m}$, and $N_{f}$ represent the total number of individuals, the number of males, and the number of females, respectively.

(2) $$N_{m}\approx \frac{N}{2}$$

(3) $$N_{f}=N-N_{m}$$

During this phase, the optimal male $f_{\text{best},m}$ and female $f_{\text{best},f}$ within their respective groups are identified, and the position of the food $f_{\text{food}}$ is established. The temperature (*Temp*) and the quantity of food (*Q*) are determined according to Eqs. (4), (5), where *t* denotes the current iteration, *T* signifies the maximum iteration, and $c_{1}$ is a constant set at 0.5.

(4) $$Temp=e^{-\left(\frac{t}{T}\right)}$$

(5) $$Q=c_{1}e^{\left(\frac{t-T}{T}\right)}$$

##### 2.1.1.2 Exploration

If Q<Threshold1, which is set at 0.25, the snakes will initiate a quest for food by choosing a random position and adjusting it accordingly. The exploration process is formulated as follows:

(6) $$X_{i,m}\left(t+1\right)=X_{rand,m}\left(t\right)\pm c_{2}A_{m}\left(\left(rand\right)\left(X_{\max}-X_{\min}\right)+X_{\min}\right)$$

Here, $X_{i,m}$, $X_{rand,m}$ and $A_{m}$ denote the position of the $i^{th}$ male, a randomly chosen male position, and the male's capability to locate food, respectively. *rand* is a randomly generated number within the range of 0–1, and $c_{2}$ is a constant set to 0.05.

(7) $$A_{m}=e^{-\left(\frac{f_{rand,m}}{f_{i,m}}\right)}$$

here, $f_{rand,m}$ and $f_{i,m}$ denote the fitness of $X_{rand,m}$ and $i^{th}$ individual in the male group, respectively.

(8) $$X_{i,f}\left(t+1\right)=X_{rand,f}\left(t\right)\pm c_{2}A_{f}\left(\left(rand\right)\left(X_{\max}-X_{\min}\right)+X_{\min}\right)$$

here, $X_{i,f}$, $X_{rand,f}$ and $A_{f}$ denote the $i^{th}$ female position, random female position, and female's capability to locate food, respectively.

(9) $$A_{f}=e^{-\left(\frac{f_{rand,f}}{f_{i,f}}\right)}$$

here, $f_{rand,f}$ and $f_{i,f}$ represent the fitness of $X_{rand,f}$ and $i^{th}$ individual in the female group, respectively.

##### 2.1.1.3 Exploitation

If Q>Threshold2, the events take place as follows:

When the temperature exceeds the *Threshold2*, which is 0.6 and is deemed hot, the snakes exclusively navigate towards the food source.

(10) $$X_{i,j}\left(t+1\right)=X_{food}\pm c_{3}\left(Temp\right)\left(rand\right)\left(X_{food}-X_{i,j}\left(t\right)\right)$$

here, $X_{i,j}$ and $X_{food}$ denote the position of an individual male or female and the position of the best individuals, respectively. The constant $c_{3}$ is set to a value of 2.

If Temp<Threshold2 criteria is met, which is considered cold, the snakes go into fight mode or mating mode.

#### 2.1.2 Fighting mode

(11) $$X_{i,m}\left(t+1\right)=X_{i,m}\left(t\right)\pm c_{3}FM\left(rand\right)\left({QX}_{best,f}-X_{i,m}\left(t\right)\right)$$

Here, $X_{i,m}$, $X_{best,f}$, and *FM* represent the position of the $i^{th}$ male, the position of the best individual in the female group, and the fighting ability of the male agent, respectively.

(12) $$X_{i,f}\left(t\right)=X_{i,f}\left(t\right)\pm c_{3}FF\left(rand\right)\left({QX}_{best,m}-X_{i,f}\left(t+1\right)\right)$$

Here, $X_{i,f}$, $X_{best,m}$, and *FF* represent the position of the $i^{th}$ female, the position of the best individual in the male group, and the fighting ability of the female agent, respectively. Eqs. (14), (13) can be used to obtain *FF* and *FM*, respectively.

(13) $$FF=e^{-\left(\frac{f_{best,m}}{f_{i}}\right)}$$

(14) $$FM=e^{-\left(\frac{f_{best,f}}{f_{i}}\right)}$$

##### 2.1.2.1 Mating mode

(15) $$X_{i,m}\left(t+1\right)=X_{i,m}\left(t\right)\pm c_{3}M_{m}\left(rand\right)\left(QX_{i,f}\left(t\right)-X_{i,m}\left(t\right)\right)$$

(16) $$X_{i,f}\left(t+1\right)=X_{i,f}\left(t\right)\pm c_{3}M_{f}\left(rand\right)\left(QX_{i,m}\left(t\right)-X_{i,f}\left(t\right)\right)$$

here, $M_{m}$ and $M_{f}$ denote the capabilities of mating for male and female individuals, respectively. Eqs. (17), (18) can be used to obtain $M_{m}$ and $M_{f}$, respectively.

(17) $$M_{m}=e^{-\left(\frac{f_{i,f}}{f_{i,m}}\right)}$$

(18) $$M_{f}=e^{-\left(\frac{f_{i,m}}{f_{i,f}}\right)}$$

The hatching of an egg represents the addition of new individuals to the population. In this case, the worst male and female individuals are replaced with the new additions.

The working mechanism of the SO algorithm is summarized as a pseudocode, which is given in Algorithm 1.

**Algorithm 1:**
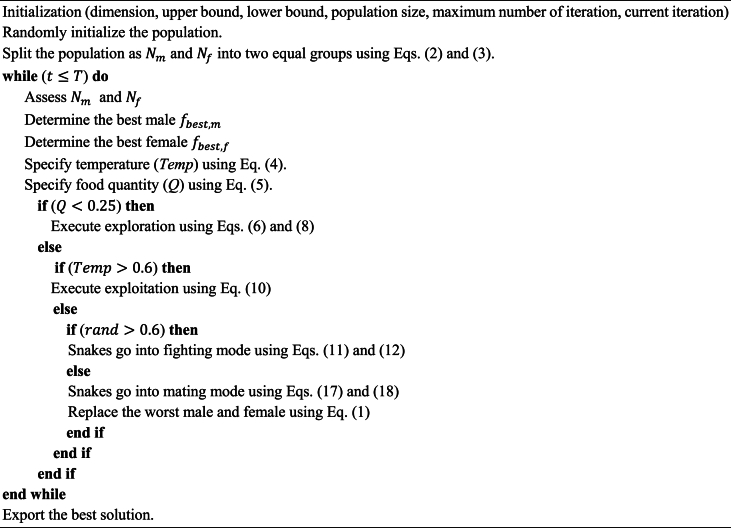
Pseudocode of the snake optimization algorithm.

### 2.2 Opposition-based learning

Opposition-Based Learning (OBL) is a methodology designed to enhance the exploration capabilities of metaheuristic algorithms. It achieves this by concurrently exploring both the current position and its opposite direction [52]. OBL involves assessing a range of candidate positions in the pursuit of the optimal solution. In scenarios where information about the distance from the current position to the best solution is lacking, the search for the optimal solution can be time-consuming. OBL addresses this challenge by leveraging both the current position and its opposite to expedite the process of identifying the best solution. The formulations for these positions are specified in Eqs. (19), (20) for one- and n-dimensional space, respectively.

(19) $$\bar{x}=U+L-x$$

(20) $${\bar{x}}_{i}=U_{i}+L_{i}-x_{i}$$

Here, *x* represents the current position, while $\bar{x}$ signifies the opposite position. The distances from the current position and its opposite to the optimal solution are continually computed and compared. The more accurate estimate is embraced at each iteration, thereby progressively advancing the search towards the optimal solution.

### 2.3 Pattern search algorithm

Pattern Search (PS) techniques entail discerning effective search point patterns from recent historical data and leveraging this knowledge to predict potentially advantageous search points in subsequent iterations. These methodologies are classified within direct search techniques, encompassing the Simplex algorithm [53]. Within this framework, Torczon [54] introduced the Multidirectional Search (MDS) algorithm in 1989 as a derivative of the pattern search approach, specifically devised for addressing unconstrained minimization problems. The MDS algorithm identifies optimal solutions by preserving the most promising preceding vertex and concurrently executing line searches in diverse directions, thus amassing valuable exploratory data. The pseudocode of the PS algorithm is given in Algorithm 2.

In this algorithm, the procedure initiates by selecting the initial simplex, denoted as $S_{0}$, along with expansion and contraction factors μ and θ. In each iteration, a search is conducted from the current optimal vertex $v_{0}^{k}$ along each of the 'n' directions established by the edges connected to $v_{0}^{k}$. The primary objective is to identify a new vertex with a function value lower than $v_{0}^{k}$. The algorithm proceeds with the reflection step if such a vertex is identified; otherwise, it proceeds with the contraction step. During contraction, the algorithm persists until the condition $f\left(c_{i}^{k}\right)<f\left(v_{0}^{k}\right)$ is satisfied. At this point, the current vertex is exchanged with $c_{i}^{k}$, which exhibits a lower function value.

In the expansion step, the algorithm computes $f\left(e_{i}^{k}\right)$ and contrasts it with $f\left(c_{i}^{k}\right)$. Depending on the outcome, the algorithm decides whether to replace $v_{i}^{k}$ with either the expansion vertex $e_{i}^{k}$ or the reflection vertex $r_{i}^{k}$. The parameters ρ, μ, and θ, governing the lengths of the steps relative to the original simplex edges, play a pivotal role in these steps. For this implementation, ρ, μ, and θ values are assigned as 1, 2, and 0.5, respectively, in accordance with Hekimoğlu [55]. Additionally, the initial step size necessary for generating the first simplex and a tolerance value critical for algorithm termination is set to 0.05 and $10^{-5}$, respectively, as prescribed in Ref. [55]. Termination of the PS algorithm transpires either when the iteration count equals the stipulated maximum iteration count (set at 50 in this instance) or when the disparity between the worst and best solutions, termed the "distance," diminishes below the tolerance value.

**Algorithm 2:**
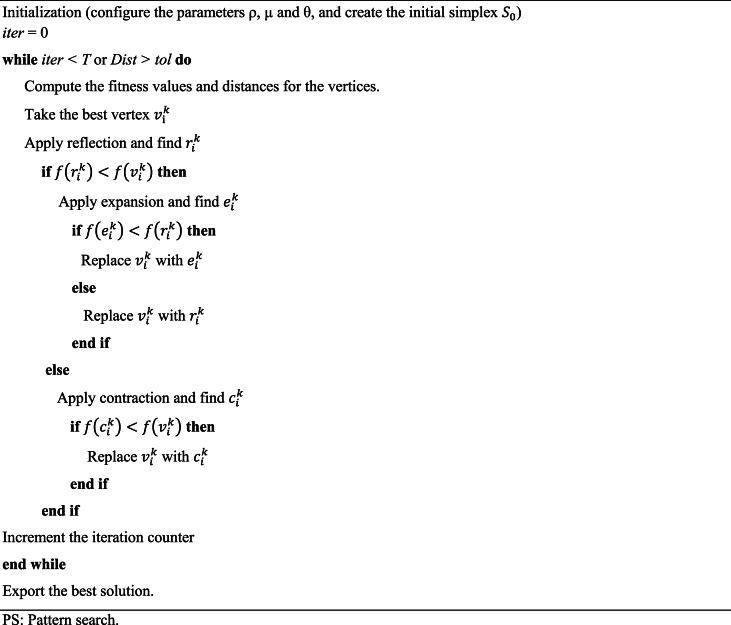
Pseudocode of the PS algorithm.

### 2.4 Proposed OSOPS algorithm

**Fig. 1:**
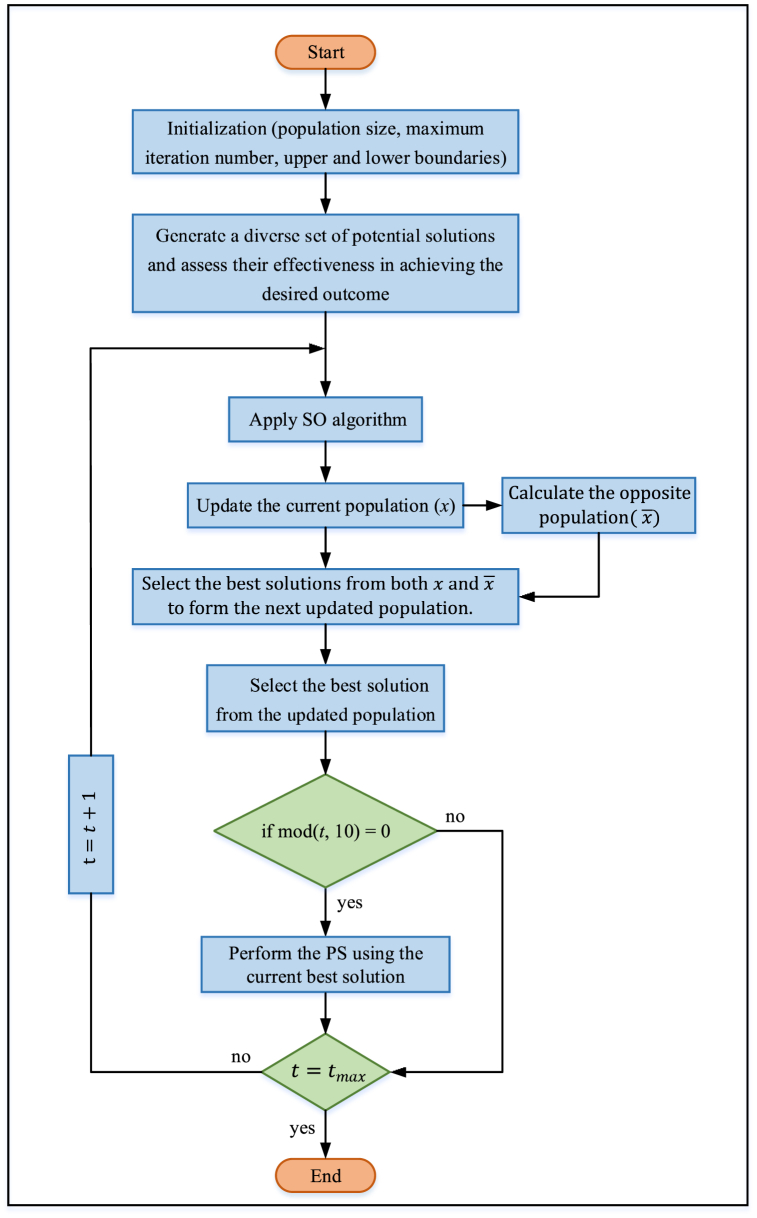
Flowchart of the OSOPS algorithm. OSOPS: Opposition-based snake optimizer with pattern search; SO: Snake optimizer; PS: Pattern search.

Although SO exhibits superior performance compared to various conventional evolutionary algorithms, as discussed in Ref. [50], there is still room for enhancement, as highlighted in Refs. [[56], [57], [58], [59]]. The integration of OBL and PS into the SO significantly enhances its performance. OBL improves the algorithm's exploration capabilities by considering the current population of solutions and their opposites. Each solution's opposite is defined concerning the problem's boundaries, which encourages exploration in different regions of the search space. This dual search strategy diversifies the search process, increasing the likelihood of escaping local optima and discovering more promising areas within the global search space, thereby effectively enhancing population diversity and exploration capabilities. On the other hand, PS is a local search technique that focuses on refining candidate solutions by exploring their immediate neighborhood. It iteratively adjusts solutions to improve their quality based on predefined patterns. Integrating PS into the OSOPS algorithm significantly enhances exploitation by fine-tuning solutions found during the exploration phase. This combination ensures that the algorithm identifies potential optimal regions and precisely optimizes solutions within these regions. The pseudocode of the proposed OSOPS algorithm is given in Algorithm 3 and Fig. 1 respectively.

When implementing the OSOPS algorithm for the non-ideal buck converter system, it is crucial to consider potential noise in the output voltage at high frequencies due to the switching nature of power electronics circuits. To address this issue, a constraint related to the open-loop crossover frequency of the system has been introduced into the optimization problem to mitigate the mentioned noise during controller design. In compliance with this constraint, the controller parameters identified by the algorithm will be disregarded, even if they offer superior performance outside the 6–12 kHz range compared to within the range. Despite the objective function potentially yielding lower values outside the 6–12 kHz range, adopting such parameters may not lead to a practical design (e.g., a system whose bandwidth is close to or exceeding the switching frequency) or not result in well-performing systems, particularly concerning disturbance rejection and signal attenuation at the switching frequency. It should be noted that this range was chosen to make a fair comparison with the system designed for a 10 kHz crossover frequency using the classical PP method. However, if desired, this range can be extended from 12 to 20 kHz.

**Algorithm 3:**
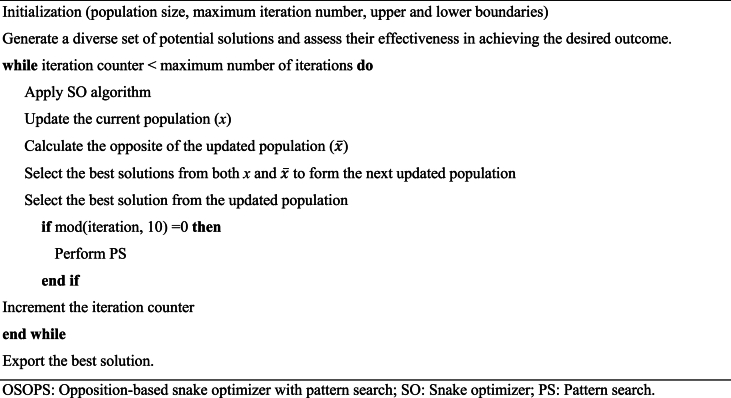
Pseudocode for proposed OSOPS algorithm.

### 2.5 Buck converter and the controller

#### 2.5.1 Mathematical model of the non-ideal buck converter

**Fig. 2:**
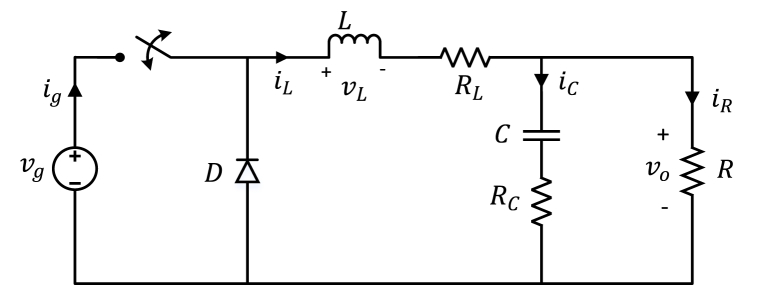
Non-ideal DC-DC buck converter.

The buck converter stands out as a prominent power electronics circuit widely utilized in various applications, including DC voltage regulation, switching power supplies, power delivery modules in computers, and DC motor drives. Its popularity stems from its straightforward implementation, uncomplicated structure, and cost-effectiveness. However, it exhibits nonlinearity [58], posing a challenge in controller design for the buck converter. Consequently, when developing a linear controller for the overall system, it becomes essential to linearize the converter. The literature commonly employs state-space averaging or circuit-averaging methods to achieve this linearization [59]. This study focuses on a non-ideal DC-DC buck converter incorporating parasitic elements, as depicted in Fig. 2.

**Fig. 3:**
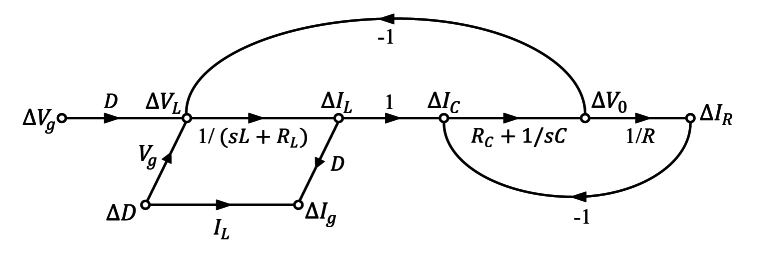
The SSFG method for deriving the small-signal model of the non-ideal DC-DC buck converter.

The switching signal-slow graph (SSFG), a technique grounded in state-space averaging, serves as a graphical representation facilitating the derivation of small-signal models for switching converters. In designing the controller, it is imperative to acquire the small-signal transfer function of the buck converter, a task accomplished through the SSFG method. The SSFG model for the non-ideal DC-DC buck converter is presented in Fig. 3. From the corresponding figure, one can derive the transfer functions for control-to-output, input-to-output, and load current-to-output, as presented in Eqs. (21), (22), (23), respectively.

(21) $$G_{V_{o}/D}\left(s\right)=\frac{\Delta V_{o}\left(s\right)}{\Delta D\left(s\right)}=\frac{{sV}_{g}RCR_{C}+V_{g}R}{s^{2}\left(RLC+R_{C}LC\right)+s\left(RCR_{L}+CR_{C}R_{L}+L+RCR_{C}\right)+R_{L}+R}$$

(22) $$G_{V_{o}/V_{g}}\left(s\right)=\frac{\Delta V_{o}\left(s\right)}{\Delta V_{g}\left(s\right)}=\frac{sDRCR_{C}+DR}{s^{2}\left(RLC+R_{C}LC\right)+s\left(RCR_{L}+CR_{C}R_{L}+L+RCR_{C}\right)+R_{L}+R}$$

(23) $$G_{V_{o}/I_{R}}\left(s\right)=\frac{\Delta V_{o}\left(s\right)}{\Delta I_{R}\left(s\right)}=-\frac{s^{2}RLCR_{C}+s\left(RCR_{L}R_{C}+RL\right)+RR_{L}}{s^{2}\left(RLC+R_{C}LC\right)+s\left(RCR_{L}+CR_{C}R_{L}+L+RCR_{C}\right)+R_{L}+R}$$

**Fig. 4:**
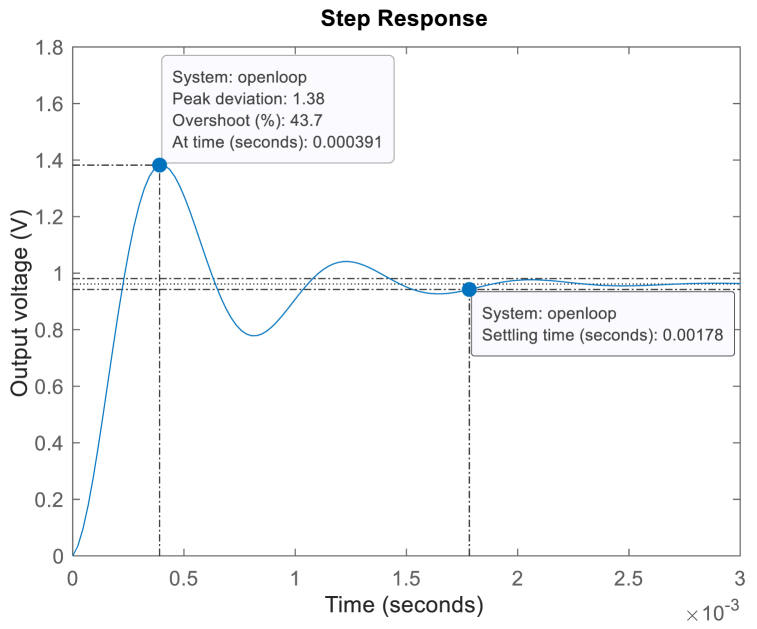
Unit step response of the uncontrolled non-ideal buck converter.

Here, *R, L, C, D,* $R_{L}$*,* $R_{C}$, $V_{g}$, and $V_{o}$, denote load, inductor, capacitor, duty cycle, inductor resistance, capacitor resistance, source voltage, and output voltage of the non-ideal buck converter, respectively. Fig. 4 illustrates the open-loop step response of the control-to-output for the non-ideal buck converter, utilizing the circuit parameter values outlined in Table 1. The asterisk in Table 1 represents the system, which is designed for the worst-case input voltage.

**Table 1:** Non-ideal buck converter parameters [60].

Parameters	Values
*R*	2.5 Ω
*L*	75 μH
*C*	220 μF
*D*	0.5*
$R_{L}$	0.1 Ω
$R_{C}$	0.07 Ω
$V_{g}$	12 V
$V_{g,worst}$	10 V
$V_{peak}$	2 V
$V_{ref}$	5 V
*f*	100 kHz

Examining Fig. 4 reveals that the unit step response deviates significantly from the ideal, displaying notable characteristics such as high overshoot, an extended settling time, and a prolonged duration to reach a steady state. Introducing a controller into the system is an effective strategy to mitigate these unfavorable transient response effects. In this context, a PID-FF controller has been selected to enhance the subpar transient response characteristics.

#### 2.5.2 PID-FF controller

**Fig. 5:**
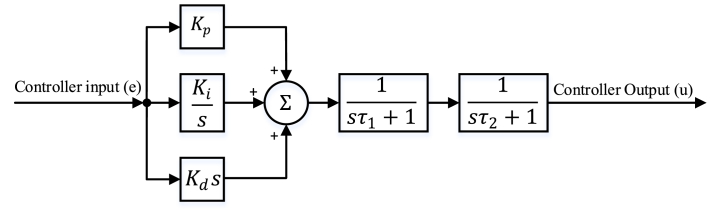
PID-FF block diagram.

A PID-FF, also known as type 3 controller, is derived by incorporating two additional cascaded first-order low-pass filters into the conventional PID controller. The first low-pass filter is used to compensate for the effect of the plant's zero, which is inherently in the transfer function of the non-ideal buck converter due to the equivalent series resistance (ESR) of the capacitor. The second one is selected such that it forces the controller gain to decrease at high frequencies and provides a sufficient gain margin and noise attenuation at the switching frequency. The bandwidth of the second filter is selected as half of the switching frequency, which is high enough that it will not inhibit the phase margin of the system [60]. Consequently, the frequencies of both low-pass filters are predetermined according to the plant, which means $\tau_{1}$ and $\tau_{2}$ coefficients in Eq. (24) are derived from those frequencies. Thus, the optimization will only be applied to PID parameters, namely, $K_{p}$, $K_{i}$, and $K_{d}$. This will provide an effective design with a less time-consuming and less complex optimization process. The block diagram of the PID-FF is illustrated in Fig. 5, and the associated transfer function is expressed in Eq. (24).

(24) $$G_{PID-FF}\left(s\right)=\left(K_{p}+\frac{K_{i}}{s}+sK_{d}\right)\left(\frac{1}{s\tau_{1}+1}\right)\left(\frac{1}{s\tau_{2}+1}\right)$$

(25) $$G_{PID-FF}\left(s\right)=\left(\frac{s^{2}K_{d}+sK_{p}+K_{i}}{s^{3}\tau_{1}\tau_{2}+s^{2}\left(\tau_{1}+\tau_{2}\right)+s}\right)$$

The pole placement (PP) method, traditionally employed to shape the closed-loop dynamics by strategically placing the poles of the system, can be applied with PID controllers to achieve enhanced control capabilities. By incorporating the pole placement technique into PID controller design, engineers can systematically tune the closed-loop response to meet specific performance requirements [[61], [62], [63], [64]]. The PID parameters for the PP method in this study are obtained using the mentioned traditional approach, which is given [64]. Here, $K_{p}$, $K_{i}$, and $K_{d}$ parameters for the PP method are calculated as 3.2884, 12800.2, and 0.0002112, respectively.

#### 2.5.3 Non-ideal buck converter with PID-FF controller

**Fig. 6:**
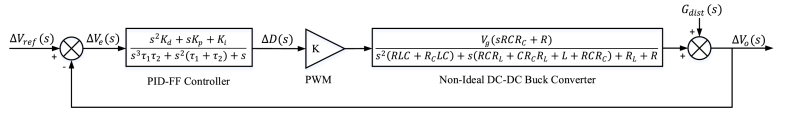
PID-FF-controlled non-ideal buck converter system.

Fig. 6 depicts the block diagram of the non-ideal buck converter incorporating the PID-FF controller. The closed-loop transfer functions of the system, accounting for the specific disturbances, can be computed using Eqs. (26), (27), (28), respectively. The gain value *K* is determined as $1/V_{peak}$, where $V_{peak}$ represents the peak amplitude of the sawtooth signal generated by the PWM block [60].

(26) $$\frac{\Delta V_{o}\left(s\right)}{{\Delta V}_{ref}\left(s\right)}=\frac{KG_{PID-FF}\left(s\right)G_{V_{o}/D}\left(s\right)}{1+{KG}_{PID-FF}\left(s\right)G_{V_{o}/D}\left(s\right)},\Delta V_{g}\;\left(s\right)\;\text{and}\;\Delta I_{R}\left(s\right)=0$$

(27) $$\frac{\Delta V_{o}\left(s\right)}{\;\Delta V_{g}\left(s\right)}=\frac{G_{V_{o}/V_{g}}\left(s\right)}{1+{KG}_{PID-FF}\left(s\right)G_{V_{o}/D}\left(s\right)},\Delta V_{ref}\left(s\right)\;\text{and}\;\Delta I_{R}\left(s\right)=0$$

(28) $$\frac{\Delta V_{o}\left(s\right)}{\Delta I_{R}\left(s\right)}=\frac{G_{V_{o}/I_{R}}\left(s\right)}{1+{KG}_{PID-FF}\left(s\right)G_{V_{o}/D}\left(s\right)},\Delta V_{g}\left(s\right)\;\text{and}\;\Delta V_{ref}\left(s\right)=0$$

here, ${\Delta V}_{ref}\left(s\right)$ and $\Delta V_{o}\left(s\right)$ denote the reference and the output voltage changes, respectively. $\Delta V_{g}\left(s\right)$ and $\Delta I_{R}\left(s\right)$, as disturbances, refer to input voltage and load current changes, respectively.

$${0.1\leq K}_{p}\leq 100$$

$${100\leq K}_{i}\leq 10^{5}$$

(29) $${10^{-6}\leq K}_{d}\leq 10^{-2}$$

The parameter boundaries for the PID-FF controller are defined by the upper and lower limits outlined in Eq. (29). These limits, akin to those presented in Ref. [60] for a type 3 controller design, have been extended to better align with the requirements of this particular study.

### 2.6 Proposed design approach

#### 2.6.1 Problem definition and objective function

Assorted metrics can function as objective criteria for the design and assessment of controllers. Commonly employed indices for minimizing error signals in controllers encompass the integral of squared error (ISE), integral of time-weighted absolute error (ITAE), integral of time-weighted squared error (ITSE), and integral of absolute error (IAE). Each of these indices has its specific advantages and drawbacks. For example, ISE provides lower overshoot and is more energy efficient; however, it exhibits a longer settling time [65]. Similarly, IAE may reduce overshoot but extend settling time, whereas ITAE can decrease both overshoot and settling time at the expense of increased computation time [66]. Taking these characteristics into account, Zwe-Lee Gaing (ZLG) [67] introduced an objective function given in (32) that minimizes maximum overshoot, steady-state error, settling time, and rise time. ZLG's superior use of transient response metrics makes it the tool of choice for regulating the output voltage of the non-ideal buck converter.

Given that the controller is designed as a voltage regulator, it will prioritize maintaining a constant output voltage for the buck converter and exhibiting robust responsiveness to variations in input voltage and load current changes. Unlike conventional designs that primarily enhance reference voltage response, this study aims to enhance the system's response to alterations in load current. By adopting this approach, disturbances are more effectively rejected, resulting in improved responsiveness to changes in input voltage, as well.

(30) $$ZLG=\left(1-e^{-E}\right)\left(M_{p}+E_{ss}\right)+e^{-E}\left(T_{s}-T_{r}\right)$$

Here, $T_{r}$, $T_{s}$, $E_{ss}$, $M_{p}$, and E denote rise time, settling time, steady-state error, maximum overshoot, and the weight coefficient, respectively. The value of E may vary across different ranges, depending on the system under analysis. An iterative trial-and-error optimization process may be employed to ascertain the appropriate value for the non-ideal buck converter system. However, here it is chosen as 1, as suggested by Ref. [60].

ZLG(x) is determined from the step response of the transfer function described in Eq. (28), with the addition of a voltage value of $R_{C}\Delta I$. This addition is necessary because, during the transition of the buck converter's output current from 1 A to 2 A, the output voltage initially decreases before gradually rising to its reference voltage. The maximum voltage drop at the output is anticipated to be $R_{C}\Delta I$ [64], and therefore, it is incorporated into the step response of Eq. (28) to facilitate the application of the objective function. It is important to note that the steady-state value of the mentioned step response, with the offset voltage, changes from 0 to $R_{C}\Delta I$. Also, to ensure the objective function is applied effectively, constrained functions of $g_{1}$ and $g_{2}$ are introduced to filter out frequencies below 6 kHz and above 12 kHz; hence, the task has become a constrained optimization problem.

#### 2.6.2 Executing the OSOPS algorithm on the non-ideal buck converter system

**Fig. 7:**
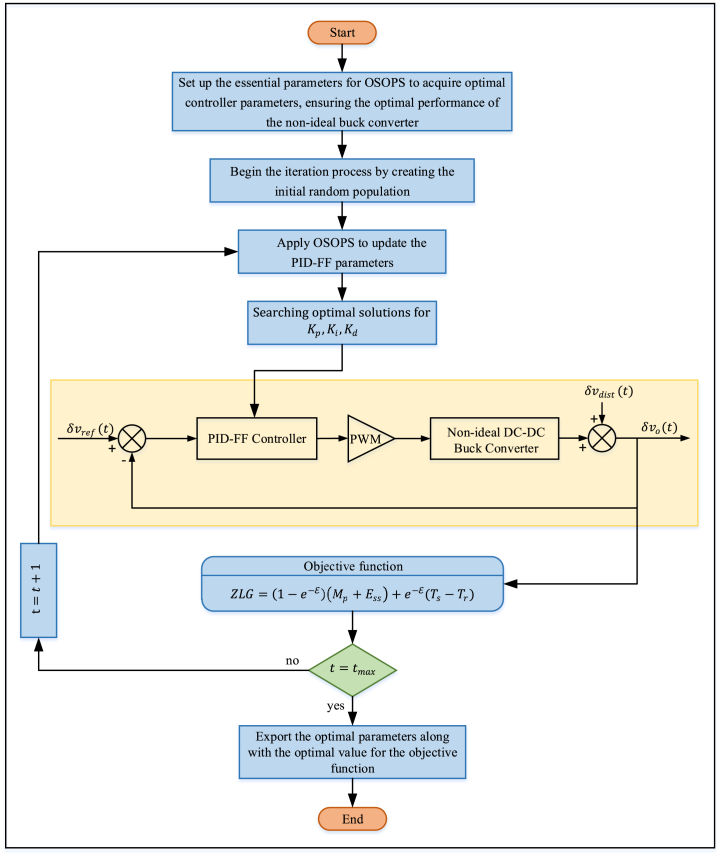
Implementation of the OSOPS to PID–FF–controlled non-ideal buck converter system. OSOPS: Opposition-based snake optimizer with pattern search; ZLG: Zwe-Lee Gaing.

Provided in Algorithm 4 is the pseudocode, and in Fig. 7 is the flowchart detailing the integration of the OSOPS methodology within the non-ideal buck converter system. The output voltage of the buck converter is governed by the PID-FF controller, the parameters of which are fine-tuned through the constrained optimization process facilitated by the OSOPS algorithm. Subsequently, the transient response metrics, including rise time, settling time, steady-state error, and overshoot of the output voltage, are employed in the computation of the objective function given in the previous subsection. The overarching goal is to minimize this objective function, thereby attaining the targeted system performance.

**Algorithm 4:**
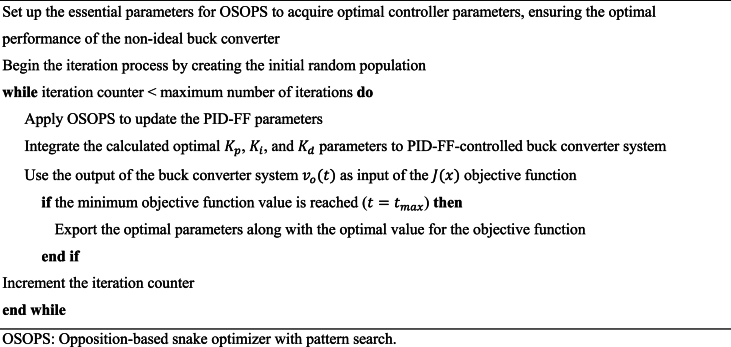
Pseudocode for the execution of the proposed OSOPS algorithm on the non-ideal buck converter system.

## 3 Results and discussion

The proposed OSOPS algorithm was assessed against the original SO algorithm [50] and classical PP [[61], [62], [63], [64]] method. The population and maximum iteration numbers were chosen to be 25 and 50, respectively, with a runtime of 30 for OSOPS and SO algorithms. Testing and evaluations were conducted on a desktop computer equipped with an Intel Core i5, 3.30 GHz processor, and 16 GB of memory. MATLAB/Simulink software was employed for the analyses mentioned above.

### 3.1 Statistical box plot analysis

**Fig. 8:**
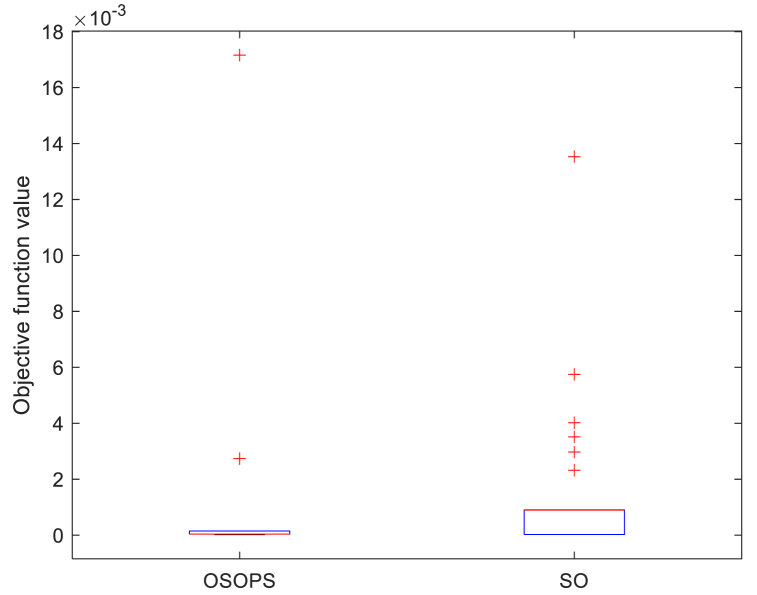
Boxplot comparison of OSOPS and SO algorithms. OSOPS: Opposition-based snake optimizer with pattern search; SO: Snake optimizer.

Table 2 displays various statistical performance metrics, including variance, standard deviation, median, mean, worst, and best. These metrics illustrate the superior performance of the OSOPS algorithm over the original SO algorithm. Additionally, a box plot analysis was employed for both OSOPS and SO to enhance the clarity of the differences, with the results depicted in Fig. 8. The comparison reveals that OSOPS exhibits lower values for minimum score, maximum score, median, upper quartile, and lower quartile compared to SO. Consequently, it can be inferred that the OSOPS algorithm outperforms the original SO algorithm.

**Table 2:** Comparison of the objective function *ZLG* between OSOPS and SO algorithms through statistical analysis.

Algorithm	Best	Worst	Mean	Median	STDEV	Var
OSOPS	1.1883 $x10^{-5}$	9.9411 $x10^{-5}$	4.4320 $x10^{-5}$	3.7374 $x10^{-5}$	2.1378 $x10^{-5}$	4.5700 $x10^{-10}$
SO	1.2103 $x10^{-5}$	343.775 $x10^{-5}$	47.6506 $x10^{-5}$	20.4493 $x10^{-5}$	71.169 $x10^{-5}$	5065.02 $x10^{-10}$

OSOPS: Opposition-based snake optimizer with pattern search; SO: Snake optimizer; STDEV: Standard deviation.

### 3.2 Convergence behaviour

**Fig. 9:**
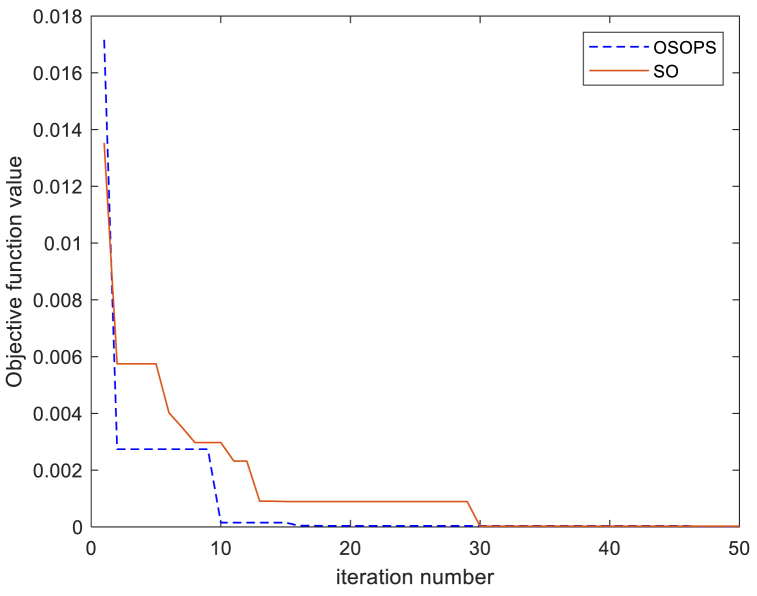
Convergence behavior of the *ZLG* objective function for OSOPS and SO algorithms. OSOPS: Opposition-based snake optimizer with pattern search; SO: Snake optimizer; ZLG: Zwe-Lee Gaing.

Fig. 9 illustrates the convergence patterns of the objective function *ZLG* derived from the execution of both the OSOPS and SO algorithms. It is crucial to acknowledge the stochastic nature of metaheuristic algorithms, leading to potential variations in results across runs. However, the OSOPS algorithm consistently outperformed SO across nearly all runs, as evidenced by the statistical box plot analysis depicted in Fig. 8. Furthermore, Fig. 9 highlights that incorporating OBL and PS methods into SO has significantly enhanced the algorithm's ability to rapidly discover improved solutions, mitigating issues such as premature convergence or stagnation in local minima.

In Table 3, one can find the optimal parameters of the PID-FF controller optimized across multiple algorithms responsible for governing the non-ideal buck converter system. It should be noted that, as mentioned in section 7.2, $\tau_{1}$ and $\tau_{2}$ are determined according to the plant's transfer function, not through an optimization algorithm. Thus, the values of $\tau_{1}$ and $\tau_{2}$ are the same for all methods given in Table 3. Furthermore, the system's simplified closed-loop transfer functions were calculated by applying the parameters acquired through the OSOPS algorithm, as specified in Equations (31), (32), (33), respectively.

(31) $$\frac{\Delta V_{o}\left(s\right)}{{\Delta V}_{ref}\left(s\right)}=\frac{2.213{x10}^{10}s^{2}+5.355{x10}^{14}s+6.958{x10}^{17}}{s^{4}+3.182{x10}^{5}s^{3}+2.345{x10}^{10}s^{2}+5.548{x10}^{14}s+6.958{x10}^{17}}$$

(32) $$\frac{\Delta V_{o}\left(s\right)}{\;\Delta V_{g}\left(s\right)}=\frac{454s^{3}+1.721{x10}^{8}s^{2}+9.261{x10}^{12}s}{s^{4}+3.182{x10}^{5}s^{3}+2.345{x10}^{10}s^{2}+5.548{x10}^{14}s+6.958{x10}^{17}}$$

(33) $$\frac{\Delta V_{o}\left(s\right)}{\;\Delta I_{R}\left(s\right)}=-\frac{0.0681s^{4}+2.591{x10}^{4}s^{3}+1.424{x10}^{9}s^{2}+91.852{x10}^{12}s+85.914}{s^{4}+{3.182{x10}^{5}s}^{3}+2.345{x10}^{10}s^{2}+5.548{x10}^{14}s+6.958{x10}^{17}}$$

**Table 3:** PID-FF parameters optimized through various approaches.

Algorithms	$K_{p}$	$K_{i}$	$K_{d}$	$\tau_{1}$	$\tau_{2}$
OSOPS	5.7829	7.5138 $x10^{3}$	0.2389 $x10^{-3}$	1.540 $x10^{-5}$	3.1831 $x10^{-6}$
SO	4.9616	6.4755 $x10^{3}$	0.2249 $x10^{-3}$	1.540 $x10^{-5}$	3.1831 $x10^{-6}$
PP	3.2884	12.8002 $x10^{3}$	0.2112 $x10^{-3}$	1.540 $x10^{-5}$	3.1831 $x10^{-6}$

OSOPS: Opposition-based snake optimizer with pattern search; SO: Snake optimizer; PP: Pattern Search.

### 3.3 Performance indices comparison

In addition to the performance metric proposed in Eq. (30), various other indices such as ISE, ITSE, IAE, and ITAE are employed to underscore the effectiveness of the OSOPS algorithm in the non-ideal buck converter system. Minimizing these indices contributes to enhanced overall stability and robustness in the controlled system, with lower values signifying increased stability. The formulations for these performance indices are presented in Eqs. (34), (35), (36), (37), respectively, where *T* is the simulation time and is set to ${1x10}^{-3}$ seconds. Table 4 reveals that the OSOPS-based system attains the lowest values across all performance indices. Consequently, the OSOPS-based system's achievement of the lowest objective function values attests to its superior performance.

(34) $$IAE=\int_{0}^{T}\left|\delta v_{ref}\left(t\right)-\delta v_{o}\left(t\right)\right|dt$$

(35) $$ISE=\int_{0}^{T}{\left(\delta v_{ref}\left(t\right)-\delta v_{o}\left(t\right)\right)}^{2}dt$$

(36) $$ITAE=\int_{0}^{T}t\left|\delta v_{ref}\left(t\right)-\delta v_{o}\left(t\right)\right|dt$$

(37) $$ITSE=\int_{0}^{T}t{\left(\delta v_{ref}\left(t\right)-\delta v_{o}\left(t\right)\right)}^{2}dt$$

**Table 4:** Performance indices comparison.

Controller type	IAE	ISE	ITAE	ITSE	ZLG
OSOPS-PID-FF	**2.6584**${x10}^{-6}$	**1.2923**${x10}^{-7}$	**7.0738**${x10}^{-11}$	**2.0012**${x10}^{-12}$	**1.6371**${x10}^{-5}$
SO-PID-FF	3.0882 ${x10}^{-6}$	1.5045 ${x10}^{-7}$	9.1877 ${x10}^{-11}$	2.6934 ${x10}^{-12}$	1.9755 ${x10}^{-5}$
PP-PID-FF	6.3117 ${x10}^{-6}$	2.1196 ${x10}^{-7}$	101.26 ${x10}^{-11}$	8.1771 ${x10}^{-12}$	480 ${x10}^{-5}$

OSOPS: Opposition-based snake optimizer with pattern search; SO: Snake optimizer; PP: Pattern Search.

### 3.4 Transient response analysis

**Fig. 10:**
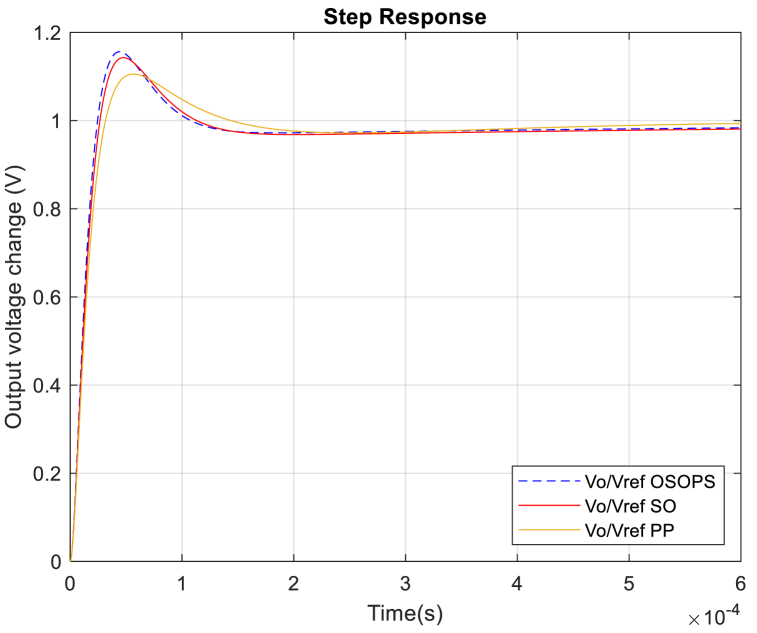
Unit step response from reference-to-output for the OSOPS and SO algorithms and the PP method. OSOPS: Opposition-based snake optimizer with pattern search; SO: Snake optimizer; PP: Pattern Search.

Fig. 10 illustrates the unit step response comparison of the reference-to-output transfer function for the non-ideal buck converter system employing a PID-FF controller based on the OSOPS and SO algorithms and the PP method. Table 5 provides a comparative analysis of the transient response performance among these systems. It should be noted that the algorithm is not designed to improve the reference-to-output transient response. As the primary objective of this study is to optimize the system for effective rejection of load current disturbances, the transient response metrics of load current-to-output emerge as the decisive factors influencing the system's performance. Thus, the results given in Fig. 12 and Table 7 are more important than the ones given here in reference-to-output response. Besides, the reference-to-output transient response represents the effect of the change in the duty cycle, so even in the worst case where the duty cycle value changes from 0 to 1 (100 %), the overshoot does not exceed 15.6 %. Considering that the duty cycle change in real-time applications is far less than 100 %, the overshoot will decrease dramatically in such applications.

**Table 5:** Reference-to-output transient response analysis of the compared algorithms.

Algorithm	Maximum overshoot (%)	Rise time (s)	Settling time (s)	Peak time (s)
OSOPS	15.60	**16.888**${x10}^{-6}$	**183.21**${x10}^{-6}$	**44.262**${x10}^{-6}$
SO	14.27	18.302 ${x10}^{-6}$	244.35 ${x10}^{-6}$	47.363 ${x10}^{-6}$
PP	**10.55**	21.215 ${x10}^{-6}$	286.59 ${x10}^{-6}$	56.406 ${x10}^{-6}$

OSOPS: Opposition-based snake optimizer with pattern search; SO: Snake optimizer; PP: Pattern Search.

### 3.5 Frequency response analysis

**Fig. 11:**
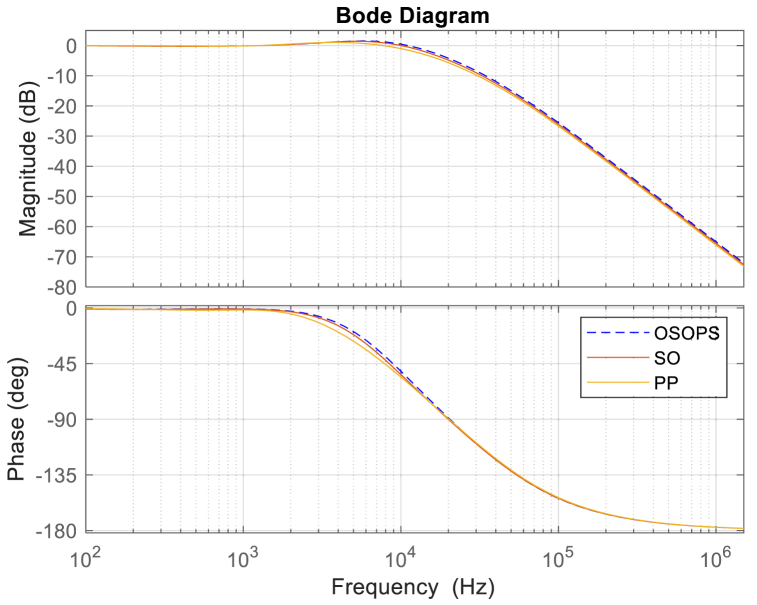
Closed-loop frequency response for the OSOPS and SO algorithms and the PP method. OSOPS: Opposition-based snake optimizer with pattern search; SO: Snake optimizer; PP: Pattern Search.

Table 6 furnishes additional details on the frequency response metrics, encompassing attenuation, crossover frequency, bandwidth, phase margin, and gain margin. The Bode plots depicted in Fig. 11 offer a comparative analysis of the performance exhibited by the proposed OSOPS and SO algorithms, along with the PP method-based non-ideal buck converter systems. The OSOPS algorithm system demonstrates a wider bandwidth than the SO- and PP-based systems. This expanded bandwidth contributes to heightened stability in the control system, as it allows for maintaining a closed-loop transfer function close to unity across a broader frequency spectrum, thereby enhancing its disturbance rejection capability. Although the OSOPS-based system may not exhibit the best attenuation performance, considering that all systems possess sufficient attenuation at the switching frequency, and given the superior performance of the OSOPS approach in diverse analyses, it is reasonable to assert that the OSOPS approach offers the most comprehensive performance for the non-ideal buck converter in this study.

**Table 6:** Frequency response analysis of the compared algorithms.

Controller type	Gain margin (dB)	Phase margin (deg)	Bandwidth (Hz)	Crossover frequency (Hz)	Attenuation at switching frequency (dB)
OSOPS	**Inf.**	61.6521	**109.70**${x10}^{3}$	**11.6**${x10}^{3}$	−26.0
SO	**Inf.**	61.8547	107.44 ${x10}^{3}$	10.9 ${x10}^{3}$	−26.5
PP	**Inf.**	**68.2731**	89.139 ${x10}^{3}$	10 ${x10}^{3}$	**−27.1**

OSOPS: Opposition-based snake optimizer with pattern search; SO: Snake optimizer; PP: Pattern Search.

### 3.6 Disturbance rejection performance

**Fig. 12:**
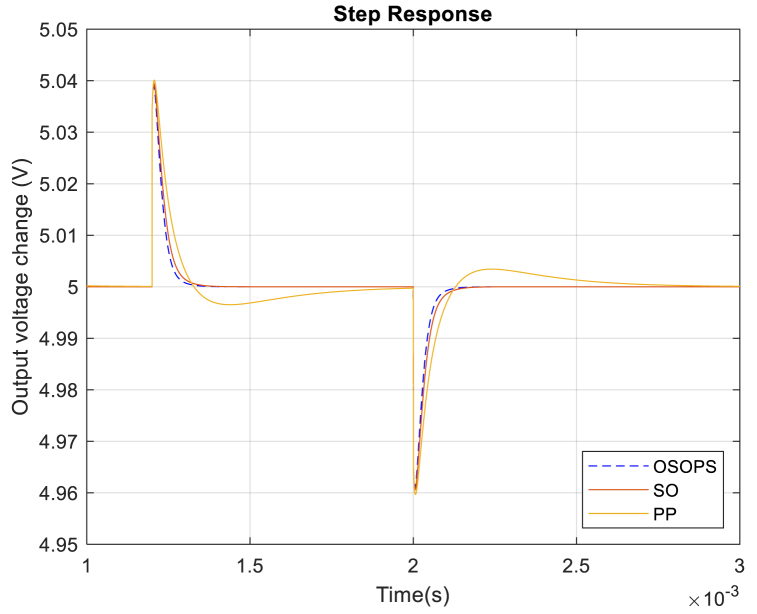
Step response of the load current disturbance rejection for the OSOPS and SO algorithms and the PP method. OSOPS: Opposition-based snake optimizer with pattern search; SO: Snake optimizer; PP: Pattern Search.

This section examines disturbances in the form of changes in load current and input voltage. Fig. 12 demonstrates how the output voltage responds to a 1 A decrease at 1.2 ms and 1 A increase at 2 ms in the load current. It is shown that the system based on OSOPS exhibits a faster disturbance rejection in comparison to alternative systems. Consequently, the proposed system is anticipated to demonstrate greater stability in real-world applications, particularly in load current disturbances scenarios.

**Fig. 13:**
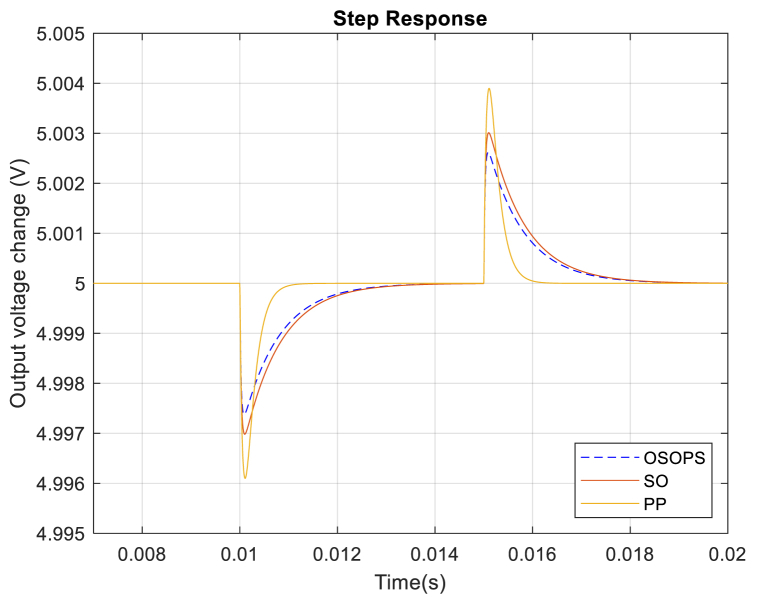
Step response of the input voltage disturbance rejection for the OSOPS and SO algorithms and the PP method. OSOPS: Opposition-based snake optimizer with pattern search; SO: Snake optimizer; PP: Pattern Search.

Likewise, Fig. 13 illustrates the step response of the system's output voltage revealing how the system responds when the input voltage decreases from 12 V to 10 V at 10 ms and increases from 10 V to 12 V at 15 ms during the simulation. Observably, the system employing the OSOPS algorithm displays the smallest overshoot compared to systems utilizing the SO and PP methods. This characteristic renders it particularly well-suited for applications subjected to uncertain conditions and characterized by variations in input voltage.

As mentioned in section 8.4, the main goal is to design the system to be most effective at load current disturbance rejection. Thus, transient response metrics of load current-to-output become the determining factor of the system performance. The results in Table 7 indicate that the non-ideal buck converter system, controlled by the PID-FF using the proposed OSOPS algorithm, exhibits the quickest rise time, settling time, and peak time compared to systems employing alternative approaches. These findings suggest that using the OSOPS algorithm in the PID–FF–controlled non-ideal buck converter system can enhance operational efficiency compared to alternative algorithms and methods investigated in this study. It is important to highlight that, typically, rise time represents the duration for a system's output to transition from 10 % to 90 %. However, the rise time values presented in Table 7 are computed from the instant the $R_{C}\Delta I$ value is introduced to the unit step response until the response reaches 90 % of its value, as the output voltage initially undergoes a decrease before the subsequent increase.

**Table 7:** Load current-to-output transient response analysis of the compared algorithms.

Algorithm	Maximum overshoot (%)	Rise time (s)	Settling time (s)	Peak time (s)
OSOPS	**0**	**6.88**${x10}^{-5}$	**11.33**${x10}^{-5}$	**18.51**${x10}^{-5}$
SO	0	8.02 ${x10}^{-5}$	13.39 ${x10}^{-5}$	21.59 ${x10}^{-5}$
PP	0.723	10.13 ${x10}^{-5}$	75.26 ${x10}^{-5}$	23.54 ${x10}^{-5}$

OSOPS: Opposition-based snake optimizer with pattern search; SO: Snake optimizer; PP: Pattern Search.

### 3.7 Robustness against parameter changes

**Fig. 14:**
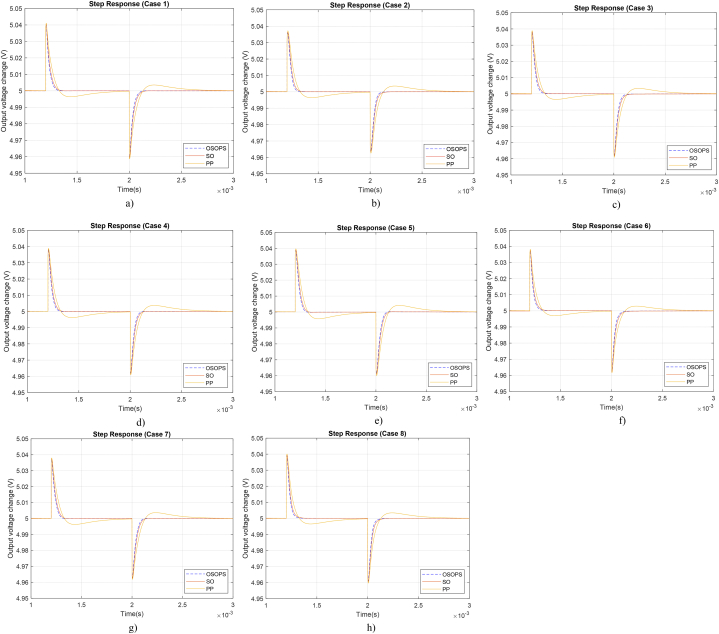
Step responses of load current disturbance rejection across different cases that are given in Table 8. OSOPS: Opposition-based snake optimizer with pattern search; SO: Snake optimizer; PP: Pattern Search.

Managing unforeseen circumstances, such as alterations in the values of plant components caused by environmental factors like temperature, humidity, and degradation over time, is a pivotal aspect of ensuring controller robustness. To assess this facet of the suggested controller, adjustments were made to the values of *L*, *C*, $R_{L}$, and $R_{C}$ within a range of plus and minus 10 %. Table 8 provides the case numbers and corresponding component values, with the altered components highlighted in bold. Each case involves modifying a single component, allowing for an examination of the specific impact induced by each component change. Fig. 14 a), b), c), d), e), f), g), and h) represent the step responses of the output voltage for cases 1, 2, 3, 4, 5, 6, 7, and 8, respectively. A careful examination reveals that, across all cases, the proposed OSOPS-based non-ideal buck converter continues to be the fastest system with the same disturbance rejection performance while exhibiting no overshoot or undershoot.

**Table 8:** Various scenarios for alterations in component values.

Case no	Capacitor resistor	Load resistor	Inductor	Capacitor
Case 1	$R_{C}=0.77\;\Omega$	$R_{L}=0.1\;\Omega$	$L=75x10^{-6}\;H$	$C=220x10^{-6}\;F$
Case 2	$R_{C}=0.63\;\Omega$	$R_{L}=0.1\;\Omega$	$L=75x10^{-6}\;H$	$C=220x10^{-6}\;F$
Case 3	$R_{C}=0.7\;\Omega$	$R_{L}=0.11\;\Omega$	$L=75x10^{-6}\;H$	$C=220x10^{-6}\;F$
Case 4	$R_{C}=0.7\;\Omega$	$R_{L}=0.09\;\Omega$	$L=75x10^{-6}\;H$	$C=220x10^{-6}\;F$
Case 5	$R_{C}=0.7\;\Omega$	$R_{L}=0.1\;\Omega$	$L=82.5x10^{-6}\;H$	$C=220x10^{-6}\;F$
Case 6	$R_{C}=0.7\;\Omega$	$R_{L}=0.1\;\Omega$	$L=67.5x10^{-6}\;H$	$C=220x10^{-6}\;F$
Case 7	$R_{C}=0.7\;\Omega$	$R_{L}=0.1\;\Omega$	$L=75x10^{-6}\;H$	$C=242x10^{-6}\;F$
Case 8	$R_{C}=0.7\;\Omega$	$R_{L}=0.1\;\Omega$	$L=75x10^{-6}\;H$	$C=198x10^{-6}\;F$

### 3.8 Trajectory reference tracking response

**Fig. 15:**
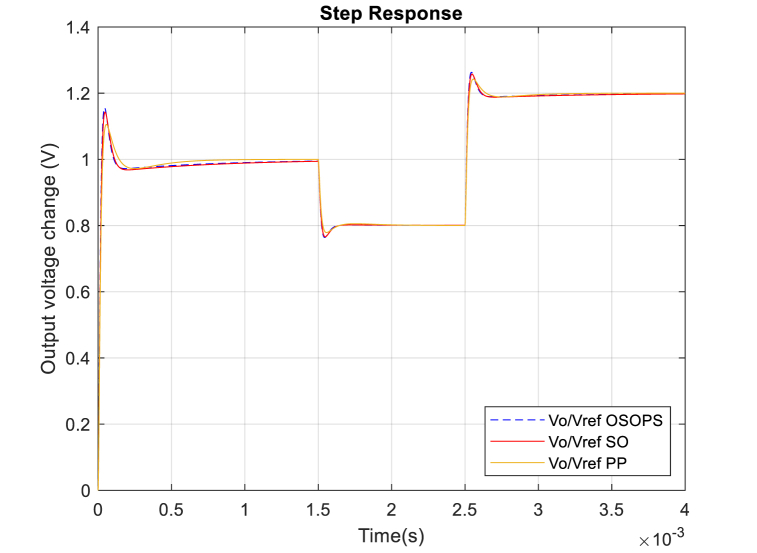
Trajectory reference tracking response from reference-to-output for the OSOPS and SO algorithms and the PP method. OSOPS: Opposition-based snake optimizer with pattern search; SO: Snake optimizer; PP: Pattern Search.

As mentioned, the proposed algorithm and the controller are designed to provide fixed output voltage and effectively reject load current and input voltage disturbances. Although the reference-to-output step response performance is not the priority here, the reference-to-output voltage disturbance response as the trajectory reference tracking response of the system is given in Fig. 15. A negative 20 % and a positive 40 % change in the reference voltage at 1.5 and 2.5 ms have been added to see the proposed algorithm-based PID-FF controller's reference-to-output step response performance. Similar performances are observed for all the compared methods.

## 4 Conclusion

An improved hybrid metaheuristic algorithm, OSOPS, is proposed to optimize the parameters of a PID-FF controller for efficient regulation of a non-ideal buck converter system. This approach combines the OBL mechanism and PS method to enhance the exploration and exploitation capabilities of the SO algorithm. The algorithm is designed to enhance the PID-FF controller parameters for effectively mitigating load current disturbances in the system, making it well-suited for real-time applications where the load voltage is regulated at a fixed value and does not change over time. Compared with the SO algorithm, the algorithm's performance has been assessed through statistical box plot analysis and convergence response analysis. The performance of the OSOPS-based PID–FF–controlled non-ideal buck converter system has been compared to the original SO algorithm and PP method-based systems, considering load current-to-output transient and frequency responses, disturbance rejection, and robustness analysis. The results show that the OSOPS-based system achieves a 14.21 % and 32.10 % faster rise time than the SO- and PP-based systems and a 15.38 % and 84.95 % faster settling time, respectively. The OSOPS and SO have higher bandwidths, surpassing the PP-based system by 18.74 % and 17.03 %, respectively. Additionally, in robustness analysis, the proposed system maintains its superior transient response with no overshoot in any of the parameter change scenarios. The proposed algorithm-based system has been shown to have the potential to be used in real-time applications where a constant voltage is required, such as supply voltages of microprocessors or drive circuits. The proposed design may also be utilized for applications where input voltage may not be constant, such as multiphase converters, renewable energy systems, switched-mode power supplies, electric vehicle chargers, uninterruptible power supplies, industrial motor drives, and HVAC systems. By demonstrating the effectiveness of the OSOPS algorithm in these various applications, future research can further validate its versatility and potential for significant advancements in control strategies across multiple domains.

Furthermore, fractional-order proportional-integral-derivative (FOPID), and FOPID with two cascaded low-pass filters (FOPID-FF) controllers, which might provide even better performance for the buck converter can be utilized for comparison with the proposed method in future studies.

However, despite these promising results, there are some limitations to this study. One limitation is the assumption that the input voltage remains constant over time, which may not be applicable in all real-world scenarios. Another one is that the integral term in the proposed controller might make the system slower, which may not be fast enough for the systems that have very fast and large amount of load change. This problem can be solved by designing a controller that can make the output impedance fully resistvive. It should be noted that the proposed algorithm can be utilized to optimize such controllers. The proposed approach may also be generalized for the systems with large amount of input voltage change. Additionally, the OSOPS algorithm's performance has been tested primarily on non-ideal buck converter systems, and its effectiveness on other types of power electronic systems remains to be explored. The computational complexity of the OSOPS algorithm is another aspect that could be investigated further, particularly in the context of real-time applications where computational resources may be limited. Future research could focus on addressing these limitations by extending the application of the OSOPS algorithm to a broader range of power electronic systems and exploring adaptive mechanisms that can handle varying load conditions. Additionally, efforts could be made to optimize the computational efficiency of the algorithm to ensure its suitability for resource-constrained environments. Further comparative studies with other state-of-the-art optimization techniques could also provide deeper insights into the relative advantages and potential areas for improvement of the OSOPS algorithm.

## Ethical statement

No ethical violations have occurred during the conception, execution, or reporting of this study. The current study was conducted independently, and no formal approval committee was involved in its execution.

## Funding

The authors received no financial support for this article's research, authorship, and publication.

## Data availability statement

No data was used for the research described in the article. Data sharing does not apply to this article.

## CRediT authorship contribution statement

**Cihan Ersali:** Writing – original draft, Investigation, Formal analysis. **Baran Hekimoglu:** Writing – original draft, Supervision, Methodology. **Musa Yilmaz:** Writing – review & editing, Project administration, Formal analysis. **Alfredo A. Martinez-Morales:** Writing – review & editing, Writing – original draft, Methodology. **Tahir Cetin Akinci:** Writing – review & editing, Software, Investigation.

## Declaration of competing interest

The authors declare that they have no known competing financial interests or personal relationships that could have appeared to influence the work reported in this paper.
